# Assessing individual-level change in dementia research: a review of methodologies

**DOI:** 10.1186/s13195-021-00768-w

**Published:** 2021-01-15

**Authors:** Aja Louise Murray, Marlena Vollmer, Ian J. Deary, Graciela Muniz-Terrera, Tom Booth

**Affiliations:** 1 Department of Psychology, University of Edinburgh, F17, 7 George Square, Edinburgh, EH8 9JZ UK; 2 Centre for Cognitive Ageing and Cognitive Epidemiology, University of Edinburgh, Edinburgh, UK; 3 Centre for Clinical Brain Sciences, Centre for Dementia Prevention, University of Edinburgh, Edinburgh, UK

**Keywords:** Alzheimer’s, Dementia, Reliable change, Individual-level change, Item response theory

## Abstract

**Background:**

Whether in the context of monitoring disease progression or in assessing the effects of interventions, a major challenge in dementia research is determining when an individual has undergone meaningful change in symptoms and other relevant outcomes such as cognitive test performance. The challenge lies in differentiating genuine improvement or deterioration from change in scores due to random and systematic error.

**Body:**

In this review, we discuss the advantages and limitations of available methods for assessing individual-level change in the context of key challenges, including imperfect and differential reliability of scores, and practice effects. We discuss indices of reliable change and the use of composite and item response theory (IRT) scores.

**Conclusion:**

We conclude that IRT-based approaches hold particular promise because they have the flexibility to accommodate solutions to a wide range of issues that influence the accuracy of judgements of meaningful change. We close by discussing the practical implications of adopting IRT-based approaches.

## Introduction

Dementia is a major public health concern, expected to only increase in importance with the estimated 35.6 million people living with dementia in 2010 projected to double by 2030 [1]. Determining whether an individual has undergone meaningful change in symptoms over time is a core task in dementia research and clinical practice; however, it is also subject to a number of challenges. These include differentiating meaningful change from change due to measurement error, practice effects, and other sources of bias and imprecision. In this review, we discuss the various methods that have been proposed to address these challenges. We highlight their strengths and weaknesses and provide recommendations for best practices and future research.

### The importance of measuring change in dementia

Determining when and for whom meaningful changes in symptoms have occurred is a key task in dementias and ageing research and in clinical practice. Evidence that meaningful cognitive decline has occurred is central in the diagnosis of dementia and mild cognitive impairment (MCI [2]). Though traditionally inferred based on estimates of premorbid functioning, clinicians are now increasingly utilising repeated assessments to determine whether an individual’s decline over time represents a steeper trajectory than would be expected as part of the normal ageing process [3]. Similarly, identifying when meaningful change has occurred is important for tracking the progression of disease over time and for predicting who will develop dementia in the future. It can thus also contribute to provision and care planning [4, 5].

Establishing when meaningful individual-level change has occurred is also necessary in the context of evaluating interventions for dementia, as well as for supporting healthy ageing more broadly. Recent decades have seen considerable efforts invested in developing and evaluating treatments for dementia and its sequelae [6, 7]. Data from randomised controlled trials can be analysed using methodologies such as multi-level models with time-by-group interaction (control versus treatment, pre- versus post-treatment) parameters or similar methodologies that compare changes in treatment and control conditions (e.g. [8]). These statistical methodologies provide information on whether, as a whole, treatment groups have shown statistically significant improvements (or attenuated declines) compared with control groups. While this is valuable information regarding the efficacy of a treatment, it does not identify whether, and which, individual trial participants have shown meaningful change. Demonstrating that meaningful change has occurred at the level of *individuals* has been argued by several authors to be the true measure of an intervention’s success because statistically significant effects in group-level statistics do not imply that any one individual has undergone clinically meaningful change [9]. Further, examining who at the individual-level has undergone clinically significant change provides an entry point for exploring sources of heterogeneity in treatment response. It may be particularly valuable in the context of adaptive trials, which utilise information on an ongoing basis for the purposes of maximising trial efficiency, as well as for early detection and enrolment of participants, including those in the prodromal phase of dementia (e.g. [10]).

### Challenges in assessing meaningful change

#### Random measurement error

Assessing whether an individual has undergone meaningful change is subject to a number of challenges and necessitates addressing sources of both random and systematic error. All assessments used in dementia are subject to random measurement error with the implication that change in scores can occur merely due to random fluctuations. The poorer the reliability of the test, the larger the fluctuations that could be expected to occur randomly and the less certain a researcher or practitioner can be that an observed change in scores reflects a genuine change in the ability or symptom being measured. Further, the reliability of assessments may depend on factors that vary across individuals, including age, gender, education, ethnicity, and a person’s level of the attribute being measured [11]. For example, previous research has suggested that the Alzheimer’s Disease Assessment Scale-Cognitive (ADAS-Cog), a measure widely used in clinical trials, is less reliable in mild and moderate dementia because some respondents answer all questions correctly, creating ceiling effects on some of its components [12]. When an assessment demonstrates lower reliability at higher/lower levels of functioning, the magnitude of change required to be confident that the change is meaningful can vary depending on an individual’s ability level.

#### Practice effects

A further issue in determining meaningful change is measurement reactivity. Measurement reactivity concerns the influence of previous administration of the same test on later measurements [13]. Practice effects are a particularly problematic form of measurement reactivity, especially in the context of cognitive tests. Practice effects refer to improvements in scores (or attenuated declines) over repeated administration of a test [14]. The need to correct for practice effects would be indicated when repeated administration of a test yields an improvement in scores in a control group. For example [15], found test score improvements over a one-week test retest period in seven of nine cognitive tests they analysed. Score improvements were greatest for visuo-spatial memory and verbal learning tasks. Practice effects have also been shown to be present in screening tools used as part of clinical trials for Alzheimer’s disease [16]. Failing to correct for practice effects when merited leads to an under-estimation of decline.

Like reliability, practice effects may vary by individual. For example, individuals who are less impaired at baseline may benefit more from practice than those who are more impaired at baseline [17, 18], although the importance of differential practice effects is debated [15].

It is worth highlighting that parallel forms of tests (i.e. measures with different content that yield comparable scores) for repeated assessments have been explored as a possible solution for practice effects (e.g. [19]). While they have shown some success, they do not appear to fully eliminate the effects of practice [16, 20]. This has been attributed to the fact that exposure to the specific content of a test is only one factor contributing to practice effects; others may include refining test-taking strategy or procedural learning (e.g. [14]). Further, developing multiple parallel forms for repeated assessments and ensuring their comparability is a major challenge in its own right, significantly increasing test development and validation demands.

## Solutions to the challenges of assessing individual-level change in dementia

### Reliable change index (RCI)

The main attempts to answer the challenges of determining meaningful individual-level change have used a reliable change framework [9]. In this framework, a statistically reliable change is considered a prerequisite for meaningful change. Reliable change is determined by establishing a threshold that a change in scores must exceed in order to be considered reliable. The threshold is selected so that an improvement or deterioration that exceeds it would not be expected due to occur measurement error alone. The necessary threshold to ensure reliable change depends on the level of random error associated with the instrument used, with lower reliability instruments requiring larger changes to indicate statistically reliable changes. Jacobson and Truax [9] advocated the use of reliable change index (RCI) developed by Jacobson et al. [21] and later modified by Christensen and Mendoza [22]. Here, a reliable change is defined as a change exceeding:

1 $$ \mathrm{RCI}=\frac{x2-x1}{{\mathrm{SE}}_{\left(x2-x1\right)}} $$

where *x*1 denotes a score at time 1, *x*2 denotes a score at time 2, and SE_(*x*2 − *x*1)_ is an estimate of the standard error of the difference score *x*2 − *x*1, making RCI a standard score. SE_(*x*2 − *x*1)_ is calculated from the standard error of measurement (*S*_*E*_):

2 $$ {\mathrm{SE}}_{\left(x2-x1\right)}=\sqrt{2{\left({S}_E\right)}^2} $$

The *S*_*E*_ in turn is calculated using an estimate of the reliability (*r*_*xx*_) and standard deviation (SD) of the test scores:

3 $$ {S}_E={\mathrm{SD}}_1\sqrt{1-{r}_{xx}} $$

Based on this, an RCI is defined for a given instrument as the magnitude of change necessary for a change in scores to be considered reliable (e.g. at *p* < .05). For example, suppose an individual scores 8 at time 1 and 10 at time 2. If a test had a reliability of .90, a population standard deviation of 2 and a reliable change at *p* < .05 was desired, then the score difference required to conclude that a reliable change has occurred could be computed as follows. Substituting these values into Eqs. 1, 2, and 3:

$$ {S}_E=2\sqrt{1-.90} $$

$$ {S}_E=0.6 $$

Then:

$$ {\mathrm{SE}}_{\left(x2-x1\right)}=\sqrt{2{(0.6)}^2} $$

$$ {\mathrm{SE}}_{\left(x2-x1\right)}=0.85 $$

And finally:

$$ \mathrm{RCI}=\frac{10-8}{0.85} $$

$$ \mathrm{RCI}=2.35 $$

Our *p* < .05 implies an RCI (standardised score change) of 1.96, based on a standard normal distribution. Thus, in this instance, our hypothetical respondent has undergone reliable change.

As an alternative, one can use the values calculated above to define a 95% confidence interval against which one can consider the raw differences. In this instance, using a *p* < .05 alpha level, we would multiply ± 1.96 ×SE_(*x*2 − *x*1)_. This would result in a 95% CI [− 1.66, 1.66], with changes falling outside of this range representing reliable change.

This basic form of RCI is, however, only one of several variants available and one that, of the full range of challenges discussed above, only addresses the issue of measurement error. Further, it assumes that measurement error is uniform across individuals; an assumption we have noted is unlikely to hold in practice.

#### RCI variants

Several previous reviews have discussed the various forms of RCI relevant for dementia research [3, 5, 23]. They have in common that (like the RCI in Eq. 1) they are essentially ratios of change scores and their standard error. RCI variants can then be classified according to two major distinctions. The first is whether the numerator is a ‘simple-difference’ or a ‘predicted-difference’ score. The second is whether the denominator (the standard error) represents dispersion around the mean of change scores or around a regression line.

#### Simple-difference score RCIs

Equation 1 is a form of simple-difference score. Simple-difference score RCIs use the observed change score in the numerator. They can be corrected for practice effects by calculating the average practice effect (i.e. average change in scores due to test re-administration) and subtracting this from an individual’s change to yield their practice-corrected change (e.g. [24]). To obtain the average change score due to test re-administration, the average change should be based on a healthy sample that is not expected to show decline (or improvement for reasons such as some intervention) over the re-test period otherwise the change due to practice alone will not be correctly estimated.

To illustrate, if the average change in scores estimated in a healthy sample on our hypothetical test was + 2 points (an improvement of 2 points), then an individual who scored 9 at baseline and 8 at follow-up would have a practice-corrected change of (8 − 9) − 2 = − 3. This would be of greater magnitude than the RCI of |1.66| for our hypothetical test with a reliability of .90 and SD of 2 and could thus be considered a reliable decline. Note in this example, the raw change uncorrected for practice effects would be − 1, a difference that would not constitute a reliable change on our hypothetical test. As in this hypothetical example, decisions as to whether to correct or not correct can carry important consequences for whether a given individual’s change is considered meaningful or not. Here, we note one simple adjustment for practice effects; however, many other approaches have been suggested in the literature [25].

There are variations across methods in terms of how the denominator (the dispersion of difference scores) of simple-difference score RCIs are computed. Where available, the actual standard deviation of the difference scores in normative data can be used; however, in practice such data may not be available for a given test. As such, various methods have been proposed to estimate this quantity. This includes variants using the standard error of measurement at baseline [9], the standard error of measurement at both baseline and retest [26], and the standard error of prediction from a regression of retest on baseline scores. In practice, there is often little difference between these choices for the denominator, although using both baseline and retest information provides a better approximation to the variance of difference scores than using baseline information alone [3].

#### Predicted-difference score RCIs

Simple-difference score RCIs correct only for unreliability and practice effects and assume both are uniform across scores. Predicted-difference score RCIs address additional challenges. Predicted-difference score RCIs use a regression prediction equation from a reference sample to compute the change score (numerator) of the RCI [27–29]. Here, the change score is the difference between the observed score at time 2 and the predicted score for time 2 estimated from the time 1 score using a regression model. This method has three principal advantages over simple-difference score methods. First, regression to the mean is accounted for by the use of predicted scores from a regression. Second, the model can be specified such that practice effects can depend on time 1 scores (rather than being assumed uniform across all individuals). Third, additional predictors and their interactions can be included in the regression model in order that the effects of variables such as age, gender, education, ethnicity, and test-retest interval can also be taken into account. Usually, the denominator of a prediction-difference score RCI will be the standard error of the estimate from the regression.

### Use of RCI in dementias and ageing research

The RCI variants discussed above have been used in dementias and ageing research for some time and it is now relatively common to compute RCIs for measures used in assessing change in symptoms or broader aspects of functioning in mild cognitive impairment (MCI) and dementia. RCIs have, for example, been computed and utilised for the Mini-Mental State Examination (MMSE [30, 31] and modified MMSE (3MS [31]), the Dementia Rating Scale [32], Consortium to Establish a Registry for Alzheimer’s Disease-Neuropsychological (CERAD-NP) battery [33], the Rey Auditory Verbal Learning Test (Rey AVLT [34]), the Repeatable Battery for the Assessment of Neuropsychological Status (RBANS [35]), and several other batteries and specific assessments (see [33] for a review). RCIs are thus becoming a standard part of the reported psychometric properties of instruments and dementia research.

## Item response theory approaches to reliable change

### Overview of IRT

While the RCIs described above have represented a significant advance in establishing reliable change, they remain subject to some important limitations. First, these RCIs were developed and have been most commonly used with ‘observed’ scores, i.e. (typically unweighted) composites (averages or sums) of the set of items in a particular assessment instrument, in the numerator. When unweighted sum scores are used, they fail to fully exploit the information available from the items in assessments. In particular, they fail to take into account the fact that not all items in an assessment are equally strongly related to the attribute that they are seeking to measure and, similarly, that they are not all equally likely to be endorsed (in symptom inventories) or answered correctly (in cognitive tests). As a result, estimates of symptoms and cognitive performance tend not to be as accurate as they could be if information regarding these item properties were taken into account. It leads to situations where, for example, two individuals differing in symptom severity could be assigned the same overall score if they endorsed the same number of symptoms, even if the symptoms endorsed by one individual tended to be more impairing or indicative of a later disease stage than the other. Similarly, the use of unweighted composite scores assumes that the measurement properties of the scores are identical across time (‘longitudinal invariance’; e.g. see [36]), where, in fact, they may change as a result of developmental processes such as ageing or previous test administration (e.g. [37]).

A second major shortcoming of the traditional RCIs reviewed above is that the measurement errors of the scores used in the denominator are estimated assuming that scores are equally reliable irrespective of a person’s level on the attribute measured by the assessment. This is important because assessments cannot be expected to be equally reliable across the full range of attribute levels that they measure, owing to the fact that the items within assessments will have a peak range of attribute levels at which they can discriminate. Assessments designed for screening or diagnostic purposes are likely, for example, to have a peak reliability around diagnostic cut-off points [38]. Thus, the score estimate for individuals scoring far away from this cut-point would be estimated with less precision than those scoring just above or below this cut-point. Where score reliability differs depending on symptom severity (e.g. [12]), traditional RCIs will not provide the individual-level calibration of change thresholds required to accurately capture reliable change.

The above issues are in principle addressed by using an IRT approach to reliable change [11, 39]. IRT models (see [40] for an introduction) are latent variable models that link observed item responses to latent unobserved attributes (e.g. cognitive ability, depression, quality of life). IRT models come in various forms but a commonly used form is the 2-parameter logistic (2PL) model for items with a binary response format. The 2PL links the probability of endorsing an item/answering it correctly to underlying attribute levels using a logistic model:

4 $$ P\left(Y=1|\theta \right)=\frac{\exp \left[\alpha \left(\theta -\beta \right)\right]}{1+\exp \left[\alpha \left(\theta -\beta \right)\right]} $$

*P*(*Y* = 1| *θ*) is the probability of endorsing an item given a person’s latent attribute level, *θ*. In addition, exp.(.) refers to the exponential function, *α* is an item discrimination parameter, and *β* is an item location (also referred to as difficulty) parameter.

Item discrimination captures the strength of relation between an item and an underlying attribute measured by a test. For items with high discrimination, item scores rise more sharply with increases in attribute levels than items with low discrimination. Higher discrimination items are thus more informative about attribute levels. Item location captures the position on the latent attribute scale that a majority of individuals endorse or correctly answer the item.

**Fig. 1:**
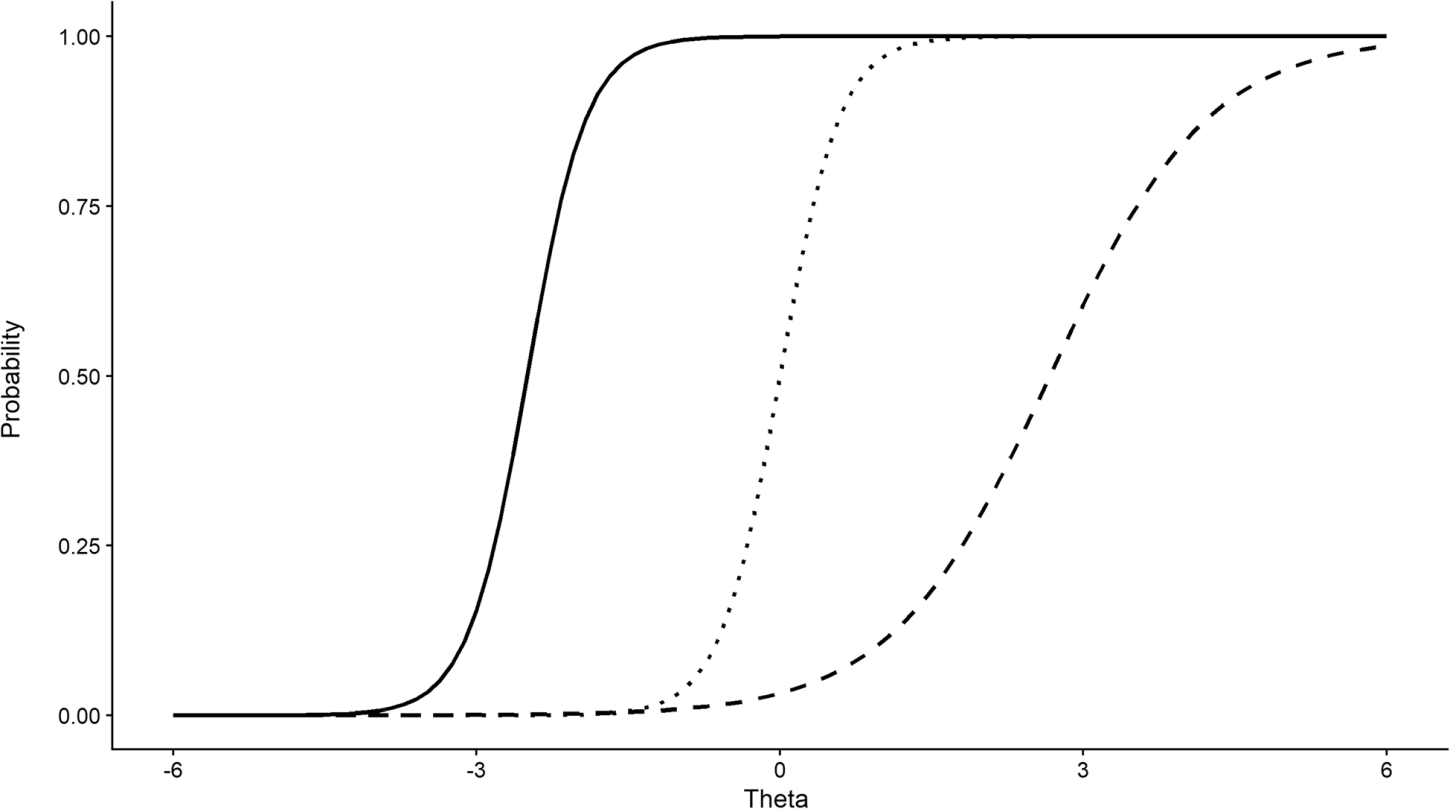
Example item characteristic curves (ICCs) for three hypothetical items. The *x*-axis shows the latent attribute (*θ*) scale. *θ* = 0 represents average cognitive ability. Negative numbers are below average *θ* levels and positive numbers are above average * θ* levels. Position on *x*-axis is determined by the location parameter and represents item difficulty. The steepness of the slopes of the lines are determined by the discrimination parameters. Items with steeper curves better discriminate between individuals with adjacent *θ* values when compared to flatter curves

These two item properties can be illuminated by examining item characteristic curves (ICC) from the 2PL. When plotted, the ICCs show the probability of endorsing an item at different attribute levels. Figure 1 shows the ICCs for three items differing in their discrimination and location parameters.

The *x*-axis shows the latent attribute scale *θ* scale. It is simplest to think of these as items of a cognitive test. Zero on the *θ* scale represents average cognitive ability. Negative numbers are below average *θ* levels and positive numbers are above average *θ* levels. The position of the curves on the *x*-axis is determined by the location parameter. Here, the item shown with the solid line would be the easiest item—that is, individuals with lower levels of  *θ* have a greater than chance (*y*-axis probability = 0.5) of getting the question correct. As we move to the right, items become harder. As such, the dashed line represents an item with the highest location (difficulty) parameter. The steepness of the lines is determined by the discrimination parameters. Here, items depicted with the solid and dotted lines have the same discrimination, but different location parameters. These items have a steeper curve, indicating that they discriminate better between individuals with similar *θ* values when compared to the third item (dashed line), which has a shallower curve.

Item characteristic curves can be summed to obtain test characteristic curves. Test characteristic curves show the relationship between the underlying attribute levels and the expected total scores on a given test and thus are useful for placing underlying levels of an attribute on the scale of the original assessment.

When extending the 2PL model to longitudinal data across two time points, it is possible to use IRT models to examine intra-individual change, analogous to approaches that have been suggested using CTT scores [41]. A first important step is to evaluate longitudinal measurement invariance. Tests of measurement invariance assess whether the measurement properties of a test are the same across time. In the context of the 2PL, these properties are the item difficulty and discrimination parameters. If measurement invariance can be established, then it can be assumed that the latent construct is equivalent at both points in time. If invariance does not hold, it is not clear that the test measures the construct in the same way at both time points. This is an important concept when studying change. If it is not clear that measurement is equivalent over time, then it is impossible to establish if any observed difference in scores reflects genuine change rather than changes in measurement. It is important to note that whenever a test of change is conducted on a simple (or weighted) sum score, measurement invariance is assumed, but not tested.

In fact, there are reasons to believe that measurement invariance over time is likely to be violated. Violations of longitudinal invariance are reasonably common in other domains (e.g. mental health) due to developmental changes in social contexts and brain development [37] and it is quite conceivable that this would also be true in ageing. For example, minor memory problems could become more noticeable to older adults if they become more attuned to signs of cognitive decline compared to their younger self, leading to differences in reporting of symptoms even in the absence of true change. Further, in the context of cognitive tests, the same problems can sometimes be solved via different strategies and older adults may shift strategies to compensate for declines in particular domains. If different abilities are drawn on to different extents to solve cognitive tests, this could also lead to violations of invariance.

Longitudinal invariance can be evaluated by comparing a set of nested models, where constraints to item location and discrimination parameters are added in sequence. In these models, correlated residuals or specific factors should be included to account for the fact that repeated measures of items will correlate with one another over and above their correlation due to their common relation with the underlying attribute being measured. In order to assess whether invariance holds, model fit comparisons are made between models with and without equivalence constraints. If model fit decreases significantly with the addition of invariance constraints, it would be concluded that invariance does not hold [42].

It is not necessary for all items to have invariant discrimination and location parameters across time provided that a subset of items (at least two but ideally more) are invariant and that the lack of invariance is modelled [43, 44]. In fact, from this point of view, baseline and retest scores need not be based on the exactly same set of items as long as a small core of items can be used to provide an ‘anchor’ that links items on to the same scale. Thus, IRT provides a framework for testing longitudinal invariance and accommodating violations of this assumption.

### IRT-based RCIs

Individual-level scores can be obtained from IRT models by treating the parameters of the model (the discrimination, difficulty, and trait correlations) as fixed and estimating scores based on these in a manner conceptually similar to deriving predicted scores from a regression model. There are several ways to estimate individual-level scores from an IRT model (see [45] for a discussion). First, they can be obtained using maximum likelihood estimation, which involves an iterative search for the set of *θ* scores that maximises the likelihood function (the product of the probabilities of all item responses). An issue with this method is that latent trait scores are not defined for some patterns of scores, for example, when an individual correctly answers all test items. Practically, such a scenario would be common when cognitive healthy individuals complete assessments such as the MMSE.

An alternative approach that resolves this issue is Bayesian estimation, where Bayesian prior information about the latent trait distribution in the population is incorporated into the estimation and a posterior distribution is formed as the product of this prior distribution and the likelihood function. The multivariate standard normal distribution is often used as the prior distribution. Methods for computing scores within this Bayesian approach include expected a posteriori (EAP) and maximum a posteriori (MAP). In these approaches, individual scores are the mean (EAP) and mode (MAP) of the posterior distribution [45].

The appropriate method of computing standard errors of measurements for individual-level scores depends on the method chosen for estimating the scores. For EAP, the standard deviation of the posterior distribution is used. For ML and MAP, they are computed as the inverse of the ‘information’ for the attribute level. Information is an IRT equivalent of reliability/precision. A distinctive and crucial feature of information and thus standard errors from an IRT perspective is that it can vary with attribute level, thus allowing for the standard error of measurement to be calibrated to an individual’s specific level.

From the IRT score estimates and their standard errors computed as described above, an IRT-based RCI can be formed. For example, Jabrayilov et al. (2016) suggests the following RCI:

5 $$ RCI=\frac{{\hat{\theta}}_2-{\hat{\theta}}_1}{\sqrt{SE{\left({\hat{\theta}}_1\right)}^2+ SE{\left({\hat{\theta}}_2\right)}^2}} $$

where ${\hat{\theta}}_1$ and ${\hat{\theta}}_2$ represent estimates of latent scores at baseline and retest respectively and $SE\left({\hat{\theta}}_1\right)$ and $SE\left({\hat{\theta}}_2\right)$ are the associated standard errors of the scores. Reise and Haviland [39] suggested a similar method whereby the 95% confidence intervals around baseline scores are calculated and a reliable change defined as occurring when the follow-up score falls outside this interval.

### Practice effects within an IRT framework

Practice effects in an IRT framework will manifest as violations of measurement invariance in the location parameters over time where items become easier to answer correctly on a second administration. Two primary solutions have been proposed [43]. First, if there is a subset of items that are resistant to practice effects over time and thus demonstrate longitudinal invariance, these items can have their parameters fixed equal over time. The remaining item parameters can vary over time to capture the effects of practice. Second, different sets of anchor items could be administered over time but their parameters fixed to known values estimated from previous studies. An appropriate reference sample can be used to estimate the item parameters. If neither approach is feasible, we suggest applying a correction directly to the attribute scores where some previous information is available on practice effects. Known practice effects on the raw scale score can be converted to the IRT score scale through the test characteristic curve. The test characteristic curve is the sum of item characteristic curves and links latent attribute levels to total scores on the test.

## A comparison of approaches to RCI calculation

In order to demonstrate the use of different forms of RCI for individual change, and the differences in substantive conclusions that may follow, here, we will provide a practical example.

### Lothian Birth Cohort 1936

Data are taken from the Lothian Birth Cohort Study 1936 (LBC1936). The LBC1936 is a longitudinal cohort study of healthy ageing, with a focus on cognitive function. Full details of the LBC1936 have been previously published [46, 47]. In brief, the study comprises, mostly, surviving members of the Scottish Mental Survey 1947, in which almost all 1936-born school children in Scottish schools took The Moray House Test No. 12 test of general intelligence test on June 4, 1947 (*N* = 70,805). Surviving members of the survey, who lived in Edinburgh (Scotland) or the surrounding area, were invited to take part in the LBC1936 study. The initial wave of testing took place in 2004–2007 with 1091 participants recruited (mean age 69.5 years, SD = 0.8 years) [46]. Participants with neurodegenerative disease at wave 1 were excluded. On entry to the study, participants were free of cognitive impairment. The normative nature of the sample makes it a good basis for estimating the IRT parameters for estimating reliable change in the NART because the sample can be assumed to be having a distribution of scores that approximately reflects that in the underlying same-aged population. However, very cognitively impaired older adults are likely to under-represented in the sample and an optimal calibration sample would include better representation of clinically impaired individuals.

For the current analysis, we make use of complete cases (*n* = 535) from waves three (mean age = 76.2 years, SD = 0.7 years) and four (mean age = 79.3 years, SD = 0.6 years) where item level responses to the National Adult Reading Test were available. In this example, we are thus considering reliable change across 3 years.

### National Adult Reading Tests

The specific data used for this example are the item responses to the National Adult Reading Test (NART [48]). The NART is a commonly used test to estimate pre-morbid intelligence in studies of ageing. Respondents are given a list of 50 irregular words and are asked to read them aloud. Words are presented in order of difficulty. For each item, respondents score 1 if the pronunciation is correct and 0 if it is incorrect. Thus, the item level data comprises 50 binary right/wrong answers at each wave.

### Analyses

RCIs were computed using three different methods.

1. A classical test theory RCI following Eq. (1). NART scores for waves 3 and 4 were simple sum scores of correct responses. The standard error was computed following Eq. (2) using the Cronbach’s alpha reliability of wave 3 NART as the estimate of *r*_xx_. Practice effects were accounted for via subtracting the mean difference score between waves from the raw difference score. A 95% confidence interval was constructed. Wave 4 scores falling outside of this interval were deemed to have undergone reliable change.

Two IRT RCIs were calculated based on a 2PL IRT model with the individual-level scores estimated using the MAP method. Scores were estimated from a longitudinal invariance model in order to address equivalence of measurement and account for practice effects via releasing parameter constraints where necessary.

2. For the first, we followed Eq. (5) above.
3. For the second, a 95% confidence interval was created based on the standard errors from wave 3, as has previously been used by [39]. Wave 4 scores outside this interval were concluded to have undergone reliable change.

In each case, we considered the number of points that would be deemed to have undergone reliable change, and also the overall agreement between the three methods. All models were estimated in R 3.6.1. The following packages were used: tidyverse(), mirt(), psych(), cowplot(), and patchwork() [49–53].

## Results

**Fig. 2:**
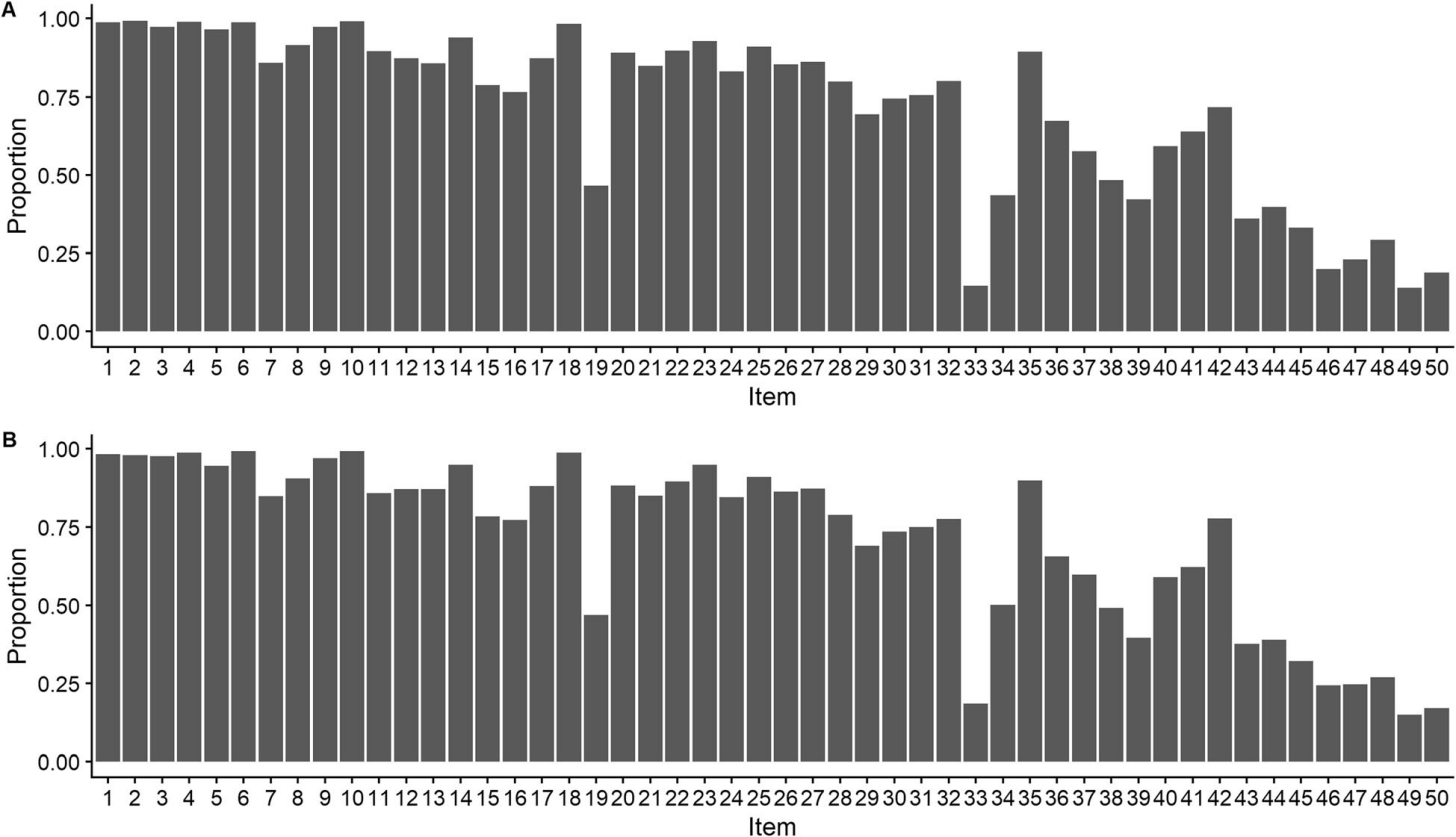
Proportion correct responses to the NART in the LBC1936 (*n* = 535) for waves 3 (**a**) and 4 (**b**)

The proportion of correct responses for the 50 NART items at waves 3 and 4 are shown in Fig. 2.

As can be seen from comparison of panels A and B in Fig. 2, on average, there is very little change in the proportion of correct responses to each item across waves. The largest difference is a 6% increase in correct answers for item 34 between waves 3 and 4.

**Fig. 3:**
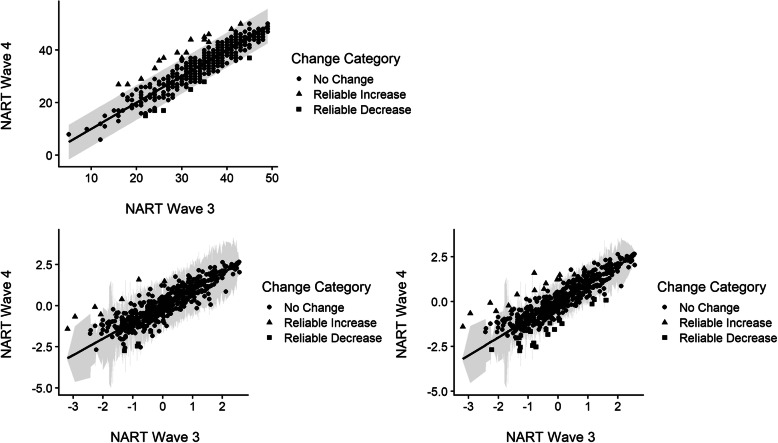
Individual change in NART scores across waves 3 and 4 in the LBC1936 estimated based on the three RCI calculation methodologies. **a** Top left: CTT method. **b** Bottom left: IRT method 1. **c** Bottom right: IRT method 2

Figure 3 shows the results of the three RCI analyses. In each plot, wave 3 scores are plotted on the *x*-axis, wave 4 scores on the *y*-axis, the diagonal line represents no change, and the shaded area shows the 95% confidence interval. Points that fall within the confidence limits are dots, points which fall outside the limits, and thus would be concluded to have undergone reliable change, are represented by squares (reliable decrease in scores) or triangles (reliable increase in scores). Tables 1, 2, and 3 show the cross-tabulation of the classification of cases depicted in Fig. 3.

**Table 1:** Cross-tabulation of CTT RCI versus IRT method 1

		IRT method 1			
		No change	Reliable increase	Reliable decrease	
CTT RCI	No change	462	8	42	512
	Reliable increase	6	10	1	17
	Reliable decrease	1	0	5	6
		469	18	48	535

**Table 2:** Cross-tabulation of CTT RCI versus IRT method 2

		IRT method 2			
		No change	Reliable increase	Reliable decrease	
CTT RCI	No change	482	3	27	512
	Reliable increase	11	6	0	17
	Reliable decrease	3	0	3	6
		496	9	30	535

**Table 3:** Cross-tabulation of IRT RCI 1 versus IRT RCI 2

		IRT method 2			
		No change	Reliable increase	Reliable decrease	
IRT method 1	No change	469	0	0	469
	Reliable increase	9	9	0	18
	Reliable decrease	18	0	30	48
		496	9	30	535

Across methods, reliable change based on the classical test theory approach classified the most participants as having undergone no reliable change. Both IRT methodologies classified more participants with reliable decreases in NART scores than the CTT approach, with method [2] being the most sensitive to the identification of reliable decreases. However, the IRT methods also classify cases as showing no change, for which the CTT method indicated either a reliable increase or decrease in scores (see first column in Tables 1 and 2). This pattern also extends to the comparison of differing IRT methods Table 3.

## Discussion

In the previous sections, we outlined various approaches that have been applied to the assessment of individual-level change in the context of challenges such as measurement error and practice effects. We noted that while traditional RCI approaches have been developed to take account of such issues, they (erroneously) assume that measurement error is equal across levels of the latent attribute and fail to fully exploit the information available from item scores. IRT scores can in principle handle all of these challenges.

The results of the empirical example presented here demonstrate that the substantive conclusions about the nature (improvement or decline), extent (magnitude), and identification of individuals who undergo reliable change varies as a function of the methodological approach taken. In the current examples, a greater number of individuals are identified as having undergone reliable change as opposed to no change, as estimated by the IRT approaches, primarily because across the range of measurement where information (reliability) is highest, the standard error of measurement decreases such that smaller magnitudes of difference indicate reliable change. Conversely, and equally important, the IRT methodologies classified some cases as having undergone no reliable change, where CTT methods indicated either a reliable increase or decrease. These observations highlight that assuming that the reliability of a test is equal across all levels of the attribute can have important implications for identifying individuals has having undergone reliable change.

In addition to allowing measurement error to vary with attribute level, other potential advantages of IRT in the context of assessing individual-level change in dementias and ageing. IRT facilitates computerised adaptive testing (CAT [54]). In CAT, each individual is administered a personalised set of items that is best calibrated to their attribute level and only until a certain precision is reached. Thus, a low functioning individual would receive easier items than a high functioning individual and each would only receive as many items as necessary to estimate their score with a pre-specified level of precision. This also means that the same participant/patient could receive items with a level of difficulty matched to their level as their disease progresses. Overall, fewer items need to be administered and participant/patient burden is minimised. Similarly, IRT facilitates linkage across datasets because provided there are a small set of anchoring items, scores from different studies can be put on a common metric [55]. This, in turn, facilitates substantially increased pooled sample sizes. Finally, IRT models can be fit for tests that measure multiple attributes. Here, measurement models such as oblique, higher-order, or bifactor models could be used to account for the structure of the test, including the correlations among the different dimensions measured by the test [56].

However, IRT models also have some disadvantages in the context of assessing individual-level change. Perhaps the main disadvantage is that IRT models are more technically and computationally demanding. While the IRT-based methods discussed above can be implemented in software such as Mplus [57] or in the mirt package in R statistical software [49], their feasibility in clinical practice may be limited. While in principle, user-friendly computerised systems could be developed, analogous to those used in traditional RCIs (e.g. [28], these currently do not exist. A second disadvantage is that simulation work suggests that IRT models appear only to have considerable advantages over observed scores when assessments contain a sufficiently large number of items. Jabrayilov et al. [11] conducted a simulation study comparing IRT and observed scores and found that IRT had no advantage in terms of detecting reliable change when the number of items was less than 20. Though the empirical example presented here concerned a test with 50 items, it is not uncommon for subscales or screening tools used in dementia to have smaller numbers of items than 20.

A further challenge for IRT approaches to individual change is the requirement that responses to individual items are available for analysis. Though electronic records of item level responses are becoming more common, it is still not always the case that such data is automatically available. In many instances, tests are still administered in paper and pencil formats by practitioners, and only total scores recorded electronically. It is not possible to utilise IRT approaches with summary data.

IRT-based RCIs also share some of the limitations of traditional RCIs. First, due to the fact that assessments used in dementia and ageing research are always subject to a degree of measurement error, a failure to identify reliable change does not imply that a change has not occurred; only that it was too small given the measurement precision of the instrument to be detected. Similarly, a reliable change does not necessarily mean that a clinically meaningful change has occurred. Clinically meaningful change must be defined according to additional standards, for example, crossing a clinical threshold or involving clinical judgement (e.g. [9]). Finally, practice effects are only one source of systematic error; there are likely to be other factors that systematically affect scores and their expected trajectory, including age, gender, ethnicity, and education, as well as, anxiety, mood, motivation, and effort at baseline and retest (e.g. [58–60]). If these factors are not taken into account, meaningful changes may be masked or true changes overstated. While it is possible to construct regression models or multi-group models that take into account these factors (e.g. IRT parameters could be allowed to vary by gender, age or education), it is difficult to measure and account for all sources of systematic variance in scores.

Another challenge in determining individual-level change is regression to the mean. Regression to the mean (e.g. [61]) concerns the distribution of a set of scores around the sample mean when data is considered across time. At any given measurement point, observed test scores will partly reflect a participant’s level of an attribute and partly reflect a random deviation from that score (‘random error’). At a single measurement point, random error can result in extreme scores that deviate substantially from an individual’s true level. However, assuming that errors are normally distributed, on second measurement, these scores are likely to be less extreme.

As retest scores will tend to gravitate towards mean scores, for those with extreme scores at baseline, regression to the mean can mask or mimic true changes over time. This means that the magnitude of change required to be confident that the change is meaningful depends on the individual (and their baseline score). Low test reliability results in greater random error and thus also greater proneness to the obscuring effects of regression to the mean. However, there remains a lack of consensus about whether and how to correct for regression to the mean in analyses of change.

Despite these limitations, balanced against their advantages, IRT-based RCIs seem to hold considerable potential for providing better estimates of reliable change in the context of research studies such as clinical trials. We identified no studies that have used IRT-based RCIs in dementia research; however, IRT methods are becoming more common in the field [55, 62]. Important future directions include further empirical evaluation and comparison of traditional versus IRT-based RCIs in both simulation studies and real data examples, the development of IRT-RCI calculators analogous to those in existence for traditional RCIs (e.g. [28]), the further development of IRT test banks to facilitate CAT testing, and the development of reference samples to provide the ‘known’ IRT parameters that can be used in methods of accounting for practice effects [43]. Finally, while the current article has focused on reliable change over two time points, the methods discussed can be extended to further time-points to compute individual-level linear and non-linear ‘slopes’ over time by combining IRT measurement models with growth curve models (e.g. [43]). Like the two-time point measures discussed here, they will require further evaluation in the context of dementia research but are likely to be useful in the increasing number of observational ageing studies with multiple ways of follow-up data.

It is also important to acknowledge that all the methods discussed typically rely on data from various cognitive assessments and psychometric tools. As such, it is important that the field continue to actively evaluate the overall quality of the measures available. From the perspective of IRT, this could include, for example, reliable range of measurement or the equivalence of scores across groups and delivery formats. Improving the quality of the measures that give rise to the scores used to evaluate change will further improve our ability to identify and characterise change in cognitive functioning.

## Conclusions

Considerable progress has been made in the development and implementation of methodologies to assess when individuals have undergone meaningful change. IRT-based RCIs hold significant promise in addressing some of the outstanding shortcomings of traditional methods, especially the dependence of measurement error on attribute level. Further empirical studies should consider adopting IRT-based RCIs as an alternative to or alongside traditional RCIs. At the same time, the field could benefit from further methodological work to further evaluate and compare different IRT-based RCIs and to make them more accessible for researchers and clinicians.

## Data Availability

The datasets generated and/or analysed during the current study are not publicly available due ethical restrictions on openly sharing the dataset. The consent forms for the study included that participants’ data, some of which is sensitive, would only be used for health-related research. Data are available by submitting a data access form to lbc1936@ed.ac.uk. R code for the analyses reported in the empirical example is available at https://osf.io/2zsu8/
